# Large aneurysmal bone cyst of iliac bone in a female child: a case report

**DOI:** 10.1186/1749-799X-5-24

**Published:** 2010-04-07

**Authors:** Anil Agarwal, Praveen Goel, Shariq A Khan, Pawan Kumar, Nadeem A Qureshi

**Affiliations:** 1 Department of Orthopedics, Chacha Nehru Bal Chikitsalaya, Geeta colony, Delhi, India

## Abstract

**Background:**

Symptomatic aneurysmal bone cysts in pediatric age group with an expansile lesion in ilium is a rare occurrence.

**Case:**

An 11-year-old female presented with a swelling over her right iliac region and numbness along the medial aspect of thigh. Clinicoradiological diagnosis was aneurysmal bone cyst confirmed on fine needle aspiration cytology. Excision curettage (wide margin excision of the soft tissue tumor and intralesional curettage in the region of acetabulum) of the tumor was performed in view of proximity to acetabular roof and endangered hip stability.

**Result:**

At follow up of 18 months, the child has full painless range of movements in the hip joint with no recurrence.

**Conclusions:**

Pelvic aneurysmal bone cysts are distinctly rare in pediatric age. The lesion was associated with an atypical symptom of numbness along the femoral nerve distribution. Hip stability and range of movements were major concern in this patient. Although many treatment options are described, surgical excision still remains the mainstay. In our case, we performed excision curettage, with good outcome.

## Background

Aneurysmal bone cysts are non-neoplastic, highly vascular, eccentric, osteolytic lesion of unknown origin that may present difficult therapeutic problems [1,2]. It's typical histological finding are blood-filled cavities lacking epithelial lining, giant cells and newly formed bony trabeculae [1]. It can occur as a primary lesion or a secondary lesion arising from other osseous conditions. Aneurysmal bone cysts are usually associated with major bone destruction, pathological fractures and local recurrence [2]. Of all aneurysmal bone cysts, about 8-12% occurs in the pelvis [1,2]. Symptomatic presentation in pediatric age group with an expansile lesion in ilium is a rare occurrence. The management of such aggressive, vascular lesion in a female child is equally challenging.

## Case report

**Figure 1:**
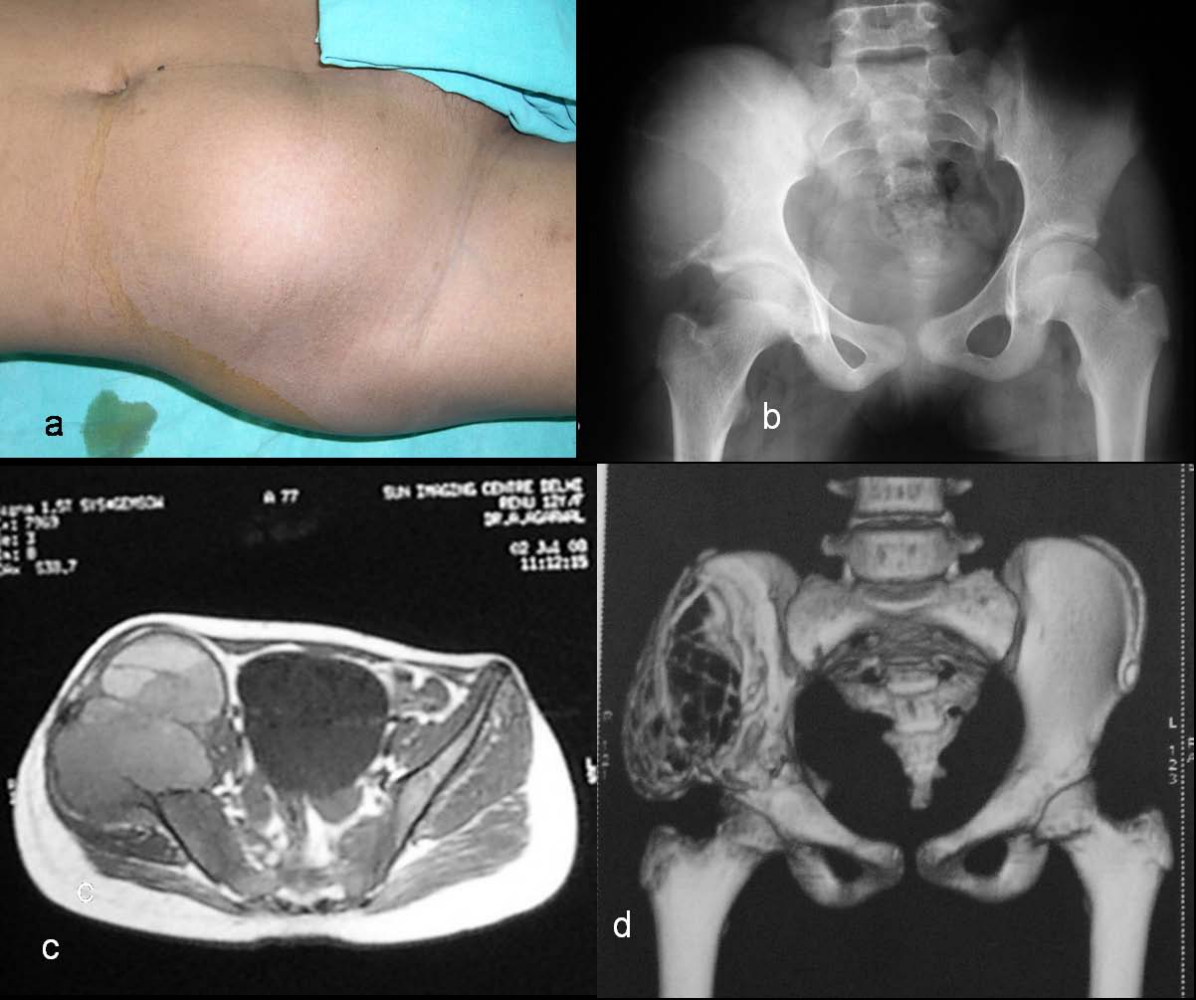
**a) Pre-operative clinical photograph showing a large swelling over the right iliac region**. b) Plain radiographs of right ilium showing involvement of the iliac wing. Multiple septations could be appreciated even on plain radiographs. c) MRI scan of right iliac region showing multiple fluid levels. d) 3D-CT reconstruction of the lesion showing a huge honeycomb appearance of lesion occupying almost whole of the right iliac wing with extension to superior acetabulum.

**Figure 2:**
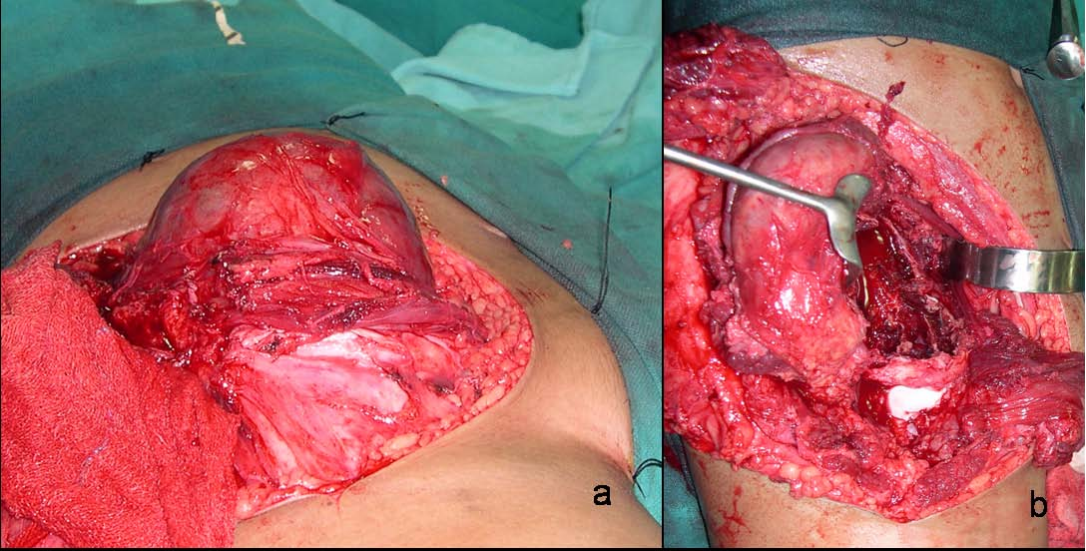
**a) Intraoperative photograph showing tumor size and the psuedocapsule**. b) Photograph after excision of lesion. Note the exposed hip joint.

**Figure 3:**
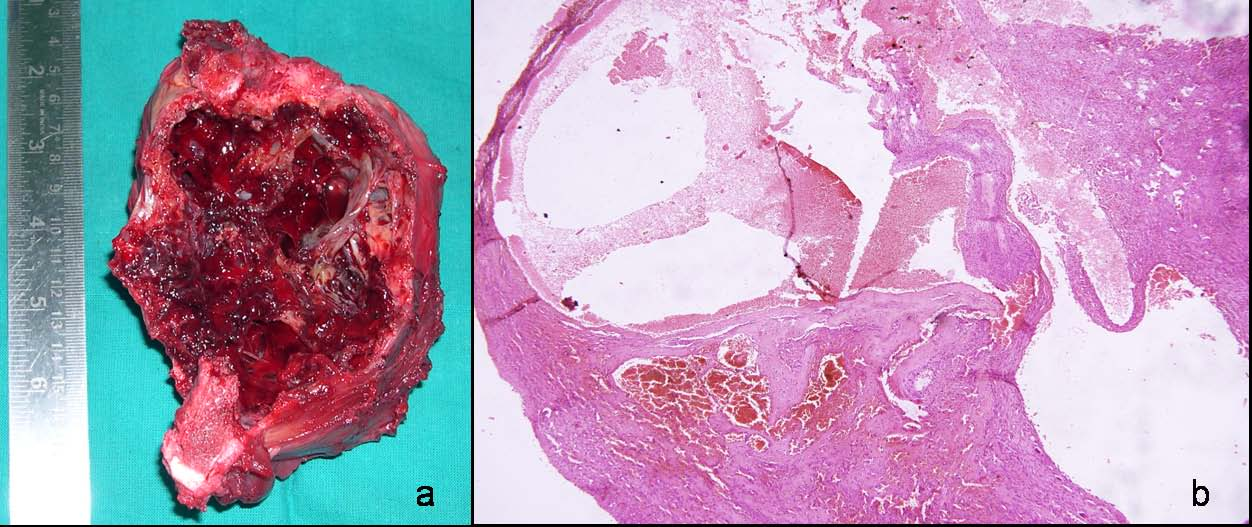
**a) Gross: The excised cyst**. b) Histopathology: Blood filled cystic spaces lined by cellular fibrous tissue lacking endothelial lining (40×; H & E staining).

**Figure 4:**
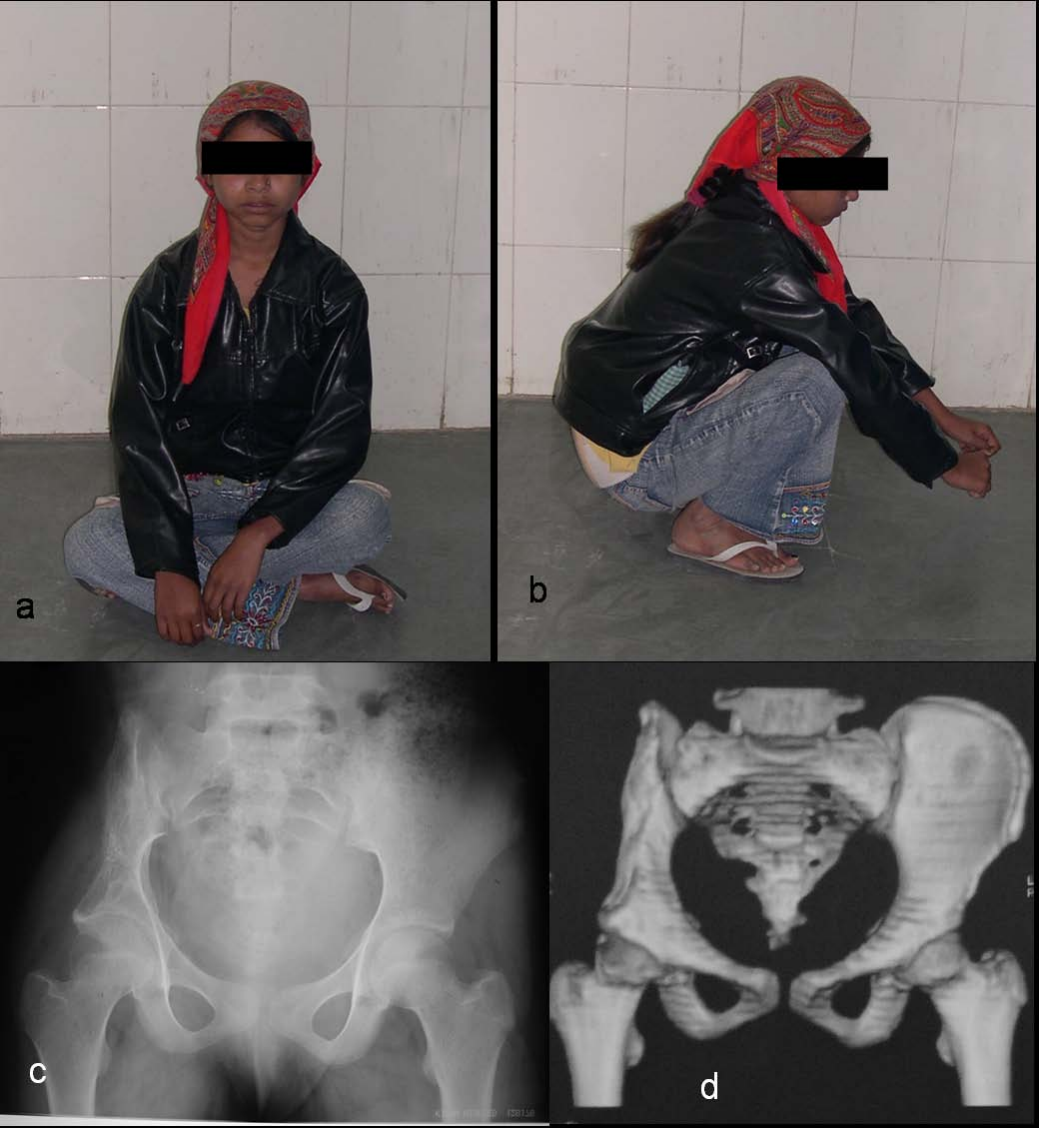
**a, b) Follow up 18 months: comfortable cross legged sitting and squatting**. c) Plain radiographs and d) CT showing good remodeling and no involvement of the hip joint.

An 11-year-old female child presented with the chief complaint of large swelling over her right iliac region which has progressively increased over a period of 4 months (Fig. 1a). She also complained of pain over her right hip region, which was dull aching, non-radiating, continuous, increased on walking, and associated with a limp. Patient walked with an antalgic gait and pointed out numbness over her right thigh which radiated along the medial aspect of thigh. There was no history of fever, any chronic illness or swellings in other body regions. Physical examination showed an approximately 16 cm × 10 cm mass over her right iliac region, which was non-movable with ill-defined margins. The swelling was warm, tender on deep palpation, and crepitations were felt over the most prominent part. Movements and power of right hip were normal except for pain during wide abduction. The neurovascular examination of right lower limb revealed hypoesthesia along medial aspect of right thigh. The blood investigations - hemogram, erythrocyte sedimentation rate, liver and renal function tests, fasting blood sugar levels, and coagulation profile were normal. Radiologically, there was an expansile cystic lesion involving the entire iliac bone from the crest to the superior border of the acetabulum with multiple septations (Fig. 1b). Magnetic resonance image (MRI) abdomen demonstrated the presence of a 14 cm × 10 cm × 8 cm large, well defined lesion, with internal septations forming cysts containing fluid levels (Fig. 1c). Computed tomography (CT) scan showed a large honeycomb type lesion of the right iliac bone extending up to the superior margin of the acetabulum, with thinned shell of cortex peripherally indicative of an expansile bone cyst (Fig. 1d). The fine needle aspiration cytology confirmed the lesion to be an aneurysmal bone cyst. The lesion was approached using a modified Smith Peterson approach. At surgery, a psuedocapsulated lesion was observed in the right iliac bone extending from the superior margin of the acetabulum to sacroiliac joint posteriorly involving almost whole of crest of ilium (Fig. 2a). The mass was noticed to produce pressure effect over the emerging femoral nerve. It was highly vascular lesion with multiple blood filled cavities. Excision curettage [2] of the tumor was performed in view of extension to the acetabular roof. In this region, the lesion was intralesionally curetted (debulked) preserving hip stability (Fig. 2b). After achieving hemostasis, the exposed hip joint and raw posterior border of the iliac bone was covered with abductor muscles. Histopathological examination of the excised mass reconfirmed the diagnosis of aneurysmal bone cyst (Fig. 3a, b). Postoperatively, she was advised complete bed rest for 4 weeks in view of the involvement of the superior margin of the acetabulum. Hip range of motion and strengthening exercises were started on the second postoperative day. By 5^th ^week, ambulation was initiated with crutch support. Four weeks later, the crutches were discarded and patient was encouraged to walk independently. At 18 months follow up, the child is an independent walker, able to squat and sit cross legged, and had full range of movements in the hip joint (Fig. 4a, b). Her abductor group has a power of 4/5 with no other neurological deficits. X-rays and enhanced CT repeated at this time showed good remodeling of the acetabulum and no signs of recurrence of the lesion (Fig. 4c, d).

## Discussion

Aneurysmal bone cysts typically involve the long bones of the extremity, membranous bones of the thorax, or vertebrae [1]. Ilium is not the site of predilection for the aneurysmal bone cysts. In the series by Papagelopoulos et al [2], the ilium bone was involved in only 8% out of 289 patients. Cottalorda et al series on 156 patients had pelvic aneurysmal bone cyst in just 9% cases [3]. Capanna detailed aneurysmal bone cysts of pelvis and mentioned four cysts that extended into ilium [4]. Other authors have mentioned involvement of iliac bone largely as case reports [1,5,6]. The only reported cases of iliac aneurysmal bone cyst in paediatric age appear mainly as part of large series of pelvic aneurysmal bone cysts or case reports [2,7,8]. Thus, a review of literature indicates that occurrence of a symptomatic aneurysmal bone cyst of ilium in pediatric age group is distinctly rare.

The method of treatment of aneurysmal bone cyst of the pelvis must be individualized depending on the location, aggressiveness and extent of the lesion. Treatment options include complete resection of the lesion, simple curettage, curettage and bone grafting, selective arterial embolization (primary treatment or preoperative adjuvant therapy) and percutaneous injection of fibrosing agent [2]. Yildirim et al [9] reported their experiences with aneurysmal bone cyst of the adult pelvis. Lesions less than 5 cm that exhibit minimal destruction or expansion of cortical bone and don't threaten the integrity of acetabulum or the sacroiliac joint are best treated with intralesional curettage, with or without bone graft. Lesion greater than 5 cm exhibiting large areas of destruction or major expansion of cortical bone and threatening the integrity of the acetabulum or the sacroiliac joint require more aggressive treatment with the use of the excision or curettage technique. Schwering et al described successful management of large iliac aneurysmal bone cyst using cystoscopic controlled curettage [8]. Chemical cauterization with phenol is recommended for relatively large primary lesion to kill any surface tumor cells of the curetted cavity [2,7,10]. Cryotherapy has also been proposed as an adjuvant therapy with surgical treatment to achieve local control [9]. Radiation is used in inaccessible sites where no surgical options are available but has high recurrence rates. Recently, percutaneous injection of fibrosing agent has been employed in the treatment of aneurysmal bone cysts. This technique is often associated with high complication rate and is expensive [9]. Selective arterial embolization is currently recommended as procedure of choice for lesions whose site or size makes other types of treatment difficult or dangerous [2]. It is especially useful for managing huge lesions posing surgical risk due to intraoperative bleeding and surrounding neural structures. The cost and availability, however, precludes its use in developing countries.

Treatment of pelvic aneurysmal bone cyst in a growing child is a challenging therapeutic problem because of the open physis, relative inaccessibility of the lesion, associated intraoperative bleeding, proximity of the lesion to neurovascular structures and the vulnerability of the acetabulum or sacroiliac joint. Stability of the hip joint was a major concern in our case, in view of the socio-cultural aspect of squatting and sitting crossed legged in the Indian setting and young age of the patient. Arthrodesis of hip joint was not acceptable to the patient's family. Marginal resection involving acetabulum would had compromised the integrity of the acetabulum and hip joint stability, hence only excision curettage of the lesion was done and sealed with surrounding muscular flaps. The integrity of the posterior ilium border and the sacroiliac joint was ensured to provide a stable hip and sacroiliac joint. Other authors have described use of autogenous tricortical iliac crest bone graft to restore the structural integrity of a compromised acetabulum [2]. Large bone defects may require reconstruction with structural allograft [2]. In few cases, where the integrity of the hip joint and the sacroiliac joint could not be preserved, drastic step of hip or sacroiliac joint fusion have been reported in the literature [2]. Adjuvant chemical cauterization was not used in our case in view of exposed hip cartilage (Fig. 2b). We could achieve excellent postoperative range of motion and a stable, pain free hip joint by preserving the acetabular roof. Cottalorda et al also expressed similar views from their experience of series of 15 pelvic aneurysmal bone cysts in children. They indicated that despite less aggressive surgical treatment in form of (intralesional) curettage, the recurrence rates are low [7].

Most of the reported recurrence of the lesion occurs within 18 months after the primary treatment [3,10]. Capanna et al in a review of 23 aneurysmal bone cysts of the pelvis treated with surgical intervention, noted a recurrence rate of 13% over a 7 years period [6]. Cottalorda et al and Papagelopoulos et al reported recurrence rate of 13% and 14% respectively [2,7]. In our case, no recurrence was noted at 18 months follow up and the iliac bone and superior margin of acetabulum had remodeled well (Fig. 4).

Iliac aneurysmal bone cysts are distinctly rare in pediatric age. The present case was a large lesion and associated with an atypical symptom of numbness along the femoral nerve distribution. Hip stability and range of movements were major concern in this patient. In our case, we performed excision curettage of the lesion with good outcome.

## Consent

Written informed consent was obtained from the patient for publication of this case report and any accompanying images. A copy of the written consent is available for review by the Editor-in-Chief of this journal.

## Competing interests

The authors declare that they have no competing interests.

## Authors' contributions

AA and SAK carried out planning and executed surgical procedure. PG, NAQ, PK participated in case follow up and drafted the manuscript. PK, PG carried out literature search. All authors read and approved the final manuscript.
